# Digital Telerehabilitation After Total Knee Arthroplasty: A Narrative Review of Clinical Outcomes, Patient Experience, Safety, and Resource Use

**DOI:** 10.26502/josm.511500276

**Published:** 2026-07-30

**Authors:** Rohan Reddy, Nicholas Lam, Mihali Dieguez, Marcel P. Fraix, Devendra K. Agrawal

**Affiliations:** 1 Departments of Translational Research, College of Osteopathic Medicine of the Pacific, Western University of Health Sciences, Pomona, California 91766 USA; 2 Physical Medicine and Rehabilitation, College of Osteopathic Medicine of the Pacific, Western University of Health Sciences, Pomona, California 91766 USA

**Keywords:** Total knee arthroplasty, Telerehabilitation, Digital health, Physical therapy, Functional recovery, Patient engagement

## Abstract

**Purpose:**

Digital telerehabilitation has become an increasingly employed form of rehabilitation therapy to support patients who have undergone total knee arthroplasty (TKA). However, various platforms differ in content, monitoring, feedback, and clinician involvement. This review summarizes recent evidence on smartphone-based and digitally mediated telerehabilitation. Functional recovery was emphasized as the primary outcome.

**Recent Findings:**

A search across PubMed, EMBASE, and CINAHL Complete was conducted from January 1^st^, 2019, to April 30^th^, 2026, resulting in eighteen postoperative TKA studies being included in the synthesis. Digital telerehabilitation generally produced functional recovery comparable to conventional physical therapy or usual care, with reported study specific advantages in range of motion, gait, strength, activity, or patient-reported function. Despite adherence and patient-experience findings often being favorable, the measures were inconsistent across studies. Additionally, pain outcomes were heterogeneous, and safety, including complications and readmissions, were limited due to incomplete reporting and low event counts. No included study provided a full economic evaluation. However, several studies reported reduced travel burden, out-of-pocket costs, or rehabilitation costs.

**Summary:**

This review integrates evidence across functional recovery, adherence, pain, satisfaction and engagement, complications and readmissions, while treating cost and resource-use findings as contextual evidence. Digital telerehabilitation appears to be a feasible adjunct or alternative for selected patients after TKA, with the clearest support for comparable functional recovery and less standardized evidence across secondary and contextual outcomes.

## 1. Introduction

Total knee arthroplasty (TKA) is an orthopedic procedure performed to reduce pain, restore function, and improve quality of life. With rising TKA procedure volumes, in part due to rising rates of obesity and older adult populations, postoperative recovery and rehabilitation have grown increasingly important [1,2]. Following TKA, rehabilitation is a key step in recovery and has traditionally been delivered through in-person physical therapy in the form of home health, hospital-based, or outpatient clinics [3]. However, shortcomings remain. Limitations consist of transportation barriers, costs, appointment availability, and shortages of health care professionals [4,5].

Progress in digital health rehabilitation has introduced additional delivery modalities to physical therapy beyond conventional in-person care. Devices such as wearable sensors, smartphone-based applications, and multimodal telerehabilitation programs seek to increase accessibility and communication with cross-disciplinary healthcare teams. These platforms may contain features such as progress monitoring, live feedback, clinician messaging, and dashboards. Through these features, digital telerehabilitation may improve adherence, convenience, and patient engagement while tackling selected challenges related to access to care [6–8].

Studies have evaluated the effectiveness, safety, usability, and cost-related findings of digital rehabilitation [2]. Evidence suggests digital rehabilitation can achieve similar or noninferior functional recovery results to conventional rehabilitation, while patient satisfaction and pain findings are less consistently reported [9]. Furthermore, selected studies have also reported outcomes beyond functional recovery, including adherence, patient experience, formal PT use, resource use, and rehabilitation costs, although these outcomes were not measured uniformly [10–12].

Despite this, the literature remains heterogeneous. Studies vary in intervention designs, technological platforms, follow-up duration, outcomes measured, as well as adherence definitions [13]. Some interventions rely heavily on smartphone and web-based education, while others incorporate wearable sensors, integrated remote monitoring, or combined models of therapist contact [14]. As a result of this, telerehabilitation is difficult to view through the lens of a single uniform intervention. Conclusive claims of superiority have yet to be established across domains such as pain outcomes, complications, readmissions, and formal cost-effectiveness evidence, which remain inconsistent or limited across current evidence bases [2].

The purpose of this review is to evaluate the effectiveness, safety, usability, and cost-related findings of smartphone-based and digitally mediated telerehabilitation compared to conventional rehabilitation or usual care following TKA. This review integrates current evidence across the domains of functional recovery, adherence, pain, satisfaction and engagement, complications and readmissions, with resource-use and cost-related findings treated as contextual evidence. Through the assessment of these domains, this investigation intends to clarify the clinical utility and relevance of digital rehabilitation in postoperative recovery after TKA.

## 2. Methods

### 2.1 Review Design and Search Strategy

This structured narrative review evaluated digitally mediated telerehabilitation after total knee arthroplasty (TKA). During screening, total knee replacement (TKR) was treated as synonymous with TKA. Telerehabilitation was defined as structured postoperative rehabilitation in which exercise, education, monitoring, feedback, or clinician communication was delivered or supported through a digital or remote platform.

PubMed, EMBASE, and CINAHL Complete were searched on April 30th, 2026. The search strategy combined three concepts: knee arthroplasty/knee replacement, digital or remote rehabilitation, and rehabilitation or physical therapy. Within each concept, the terms were combined with an OR and each concept with an AND. The searches were restricted to human and English language papers between the dates of January 1st, 2019, and the search date. At the end of the database searches (before duplicate removal) there were 137 results: 41 from PubMed, 76 from EMBASE and 20 from CINAHL.

### 2.2 Eligibility Criteria

Eligible studies included adults undergoing postoperative rehabilitation after primary TKA. Eligible interventions had to use a digital or remote platform for rehabilitation delivery, recovery monitoring, exercise guidance, adherence support, patient feedback, or clinician-directed progression. Studies in which the digital element was incidental to care and not part of the rehabilitation intervention, were not included in the main synthesis.

Eligible comparator interventions were conventional rehabilitation or home-based physical therapy, usual care, paper-based home exercise programs, or alternative telerehabilitation models. Included study types consisted of randomized trials, comparative studies, cohort studies, feasibility or pilot studies, mixed-methods studies or studies on utilization. The outcome domains of interest are functional recovery, adherence, pain, satisfaction or engagement and complications or readmissions. Findings relating to accessibility and resource use or cost were also extracted where presented but used as supplementary to the secondary domains.

Mixed total hip arthroplasty (THA)/TKA records were considered during screening when TKA-specific data appeared extractable. The main synthesis was restricted to studies with TKA-specific or knee arthroplasty results directly relevant to postoperative TKA rehabilitation. Protocols without results, case reports, commentaries, preoperative-only interventions, nonhuman or non-English records, reports without available full text, and studies without a relevant digital rehabilitation component or usable clinical outcomes were excluded from the main synthesis.

### 2.3 Study Selection:

After removal of 28 duplicate records, 109 database-derived records remained. Four records manually identified from a previous review were added, totaling 113 records for title and abstract screening. Records advanced to full-text review when the title or abstract described a postoperative TKA rehabilitation population, a digital or remote rehabilitation component, and patient-level outcomes. This process identified 45 records for full-text review, of which 37 met broad eligibility criteria and 8 were excluded because the full text proved unavailable or incomplete, the population was ineligible, the report contained abstract-only evidence, or the available full text did not meet the review criteria.

**Figure 1:**
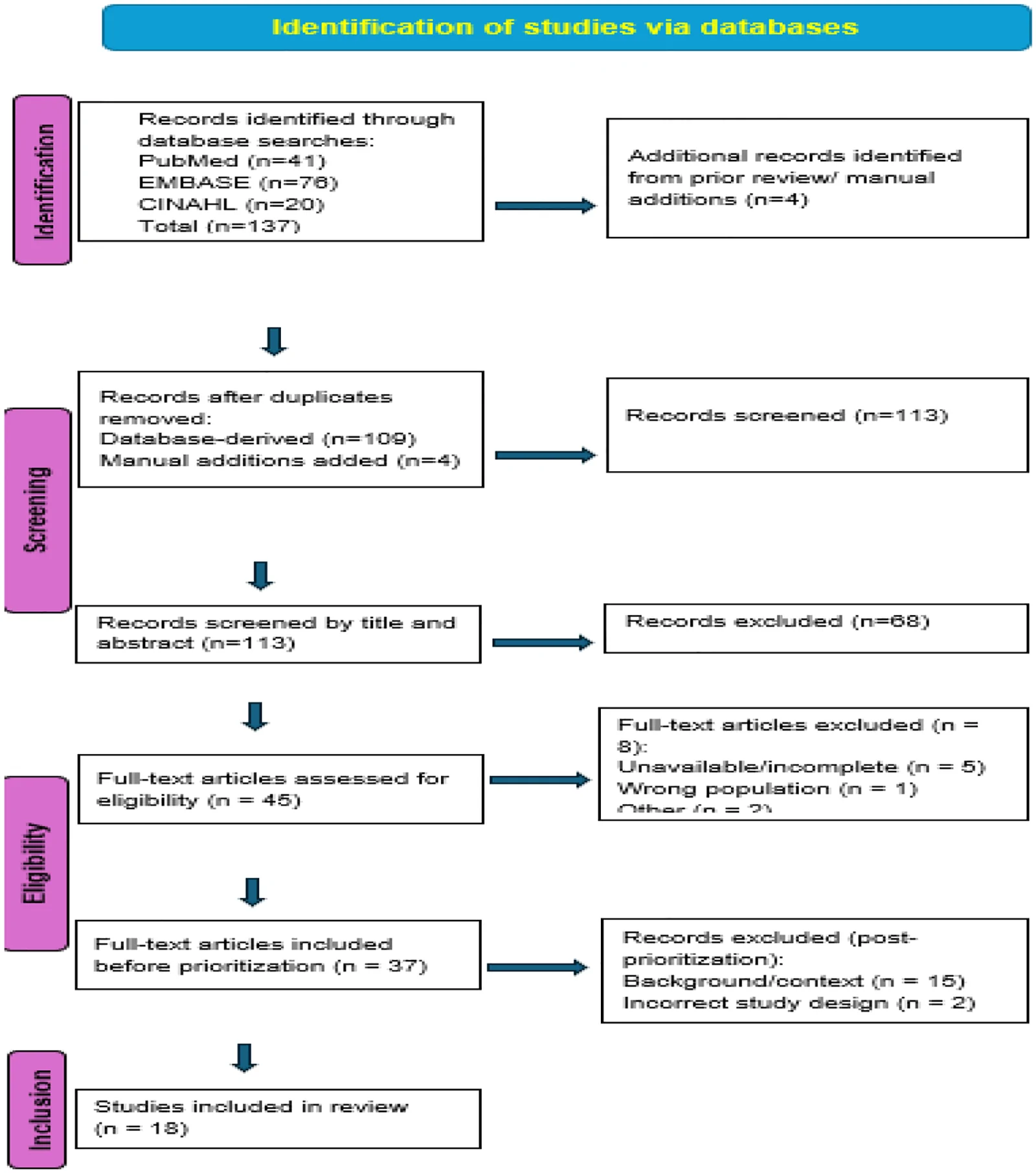
Study-selection flow diagram for article identification, screening, eligibility assessment, and main-synthesis inclusion.

The 37 eligible full-text records varied in directness to the review question. The main synthesis set was therefore limited to peer-reviewed primary studies from 2019 onward that evaluated structured digital or telerehabilitation for postoperative TKA rehabilitation or recovery and reported direct patient results. Randomized or comparative studies were emphasized. This process yielded 18 studies for the main synthesis. The study-selection process is summarized in Figure 1.

### 2.4 Data Extraction

For each study included in the main synthesis, we extracted variables related to study design, sample size, population, intervention and comparator characteristics, duration of follow-up, outcome measures, main findings and main limitations. Functional recovery was considered the primary outcome. Secondary outcomes included adherence, pain, satisfaction or engagement, and complications or readmissions. Resource use and cost data were extracted when available.

Results were grouped by outcome domain, considering study design, comparator intensity, follow-up duration, and whether the digital program was a replacement, supplement, or comparison to conventional rehabilitation or usual care.

### 2.5 Intervention Taxonomy

Interventions were grouped by the dominant way the digital platform supported rehabilitation. Wearable or sensor-supported models used wearable devices, inertial sensors, or activity tracking to guide rehabilitation. Real-time biofeedback models used motion, camera, or sensor feedback during exercise performance. Clinician-monitored or alerted models focused on remote review, dashboard alerts, a clinician alert, or direct clinician adjustment of a plan. Hybrid digitally supported models combined digitally driven intervention with synchronous communication, coaching, lifestyle advice or multimodal contact with a therapist. Because many interventions combined several components, each study was also coded for specific features such as app or web content, connected home devices, and wearable activity tracking (Table 1). A more comprehensive information of this taxonomy is provided in Table 2.

## 3. Results

### 3.1 Characteristics of Included Studies

The main synthesis included 18 studies of digitally mediated rehabilitation after total knee arthroplasty (TKA). Most used randomized or controlled comparative designs, with quasi-experimental, feasibility, and retrospective comparative designs also represented [11,15,16]. Studies were conducted throughout multiple countries, most commonly the United States and China.

Study size and follow-up varied substantially. Analyzed sample sizes ranged from fewer than 60 participants in smaller pilot studies to more than 300 participants in larger trials and comparative cohorts [10,11,17–20]. Follow-ups ranged from 12 days to 52 weeks or longer, with several studies reporting 6-month or 1-year outcomes [10,16,17, 20–22].

Seventeen studies focused on TKA, and one study enrolled both primary total knee and unicompartmental knee arthroplasty patients and was retained because TKA-relevant subgroup results were reported [10].

Eligibility criteria and comparator selection differed between studies. Some studies required a smartphone, internet, or email access [10,15,20,21,23]. Others included cognitive eligibility requirements [17,25]. Comparisons included supervised outpatient or usual physical therapy (PT) versus paper-based home exercise programs, routine follow-up, or another telerehabilitation model [13,17,19,20,21,24,25].

### 3.2 Intervention Taxonomy and Digital Components

The intervention taxonomy grouped the 18 main-synthesis studies into four digital rehabilitation models. Six studies were classified as real-time biofeedback rehabilitation [13,15,18,19,25,26], four as wearable or sensor-supported rehabilitation [10,16,20,22], five as hybrid digitally supported rehabilitation [17,24,27,29], and three as clinician-monitored or alert-enabled rehabilitation [11,21,23].

Digital services usually combined more than one rehabilitation support. App- or web-based exercise or education content was present in 17 of 18 studies, and clinician dashboard or remote monitoring features were present in 16 studies. Smart alerts and connected home rehabilitation devices were less frequent.

### 3.3 Functional Recovery

Functional recovery was the most consistently reported clinical outcome, and several higher-weight studies reported outcomes similar to comparator care [10,19–21]. Selected advantages in ROM, strength, chair-stand performance, activity, or patient-reported domains were reported in other studies [11,18,23,25–27].

Similar functional outcomes were most evident in studies utilizing formal PT or usual rehabilitation comparators. VERITAS found virtual in-home therapy using VERA to be noninferior to usual PT for 12-week KOOS, with similar knee flexion and gait speed [19]. A smartwatch/mobile app trial reported no clinically significant difference in 1-year KOOS Junior (KOOS JR) or ROM compared with formal PT, and the mymobility smartphone/smartwatch platform achieved similar 1-year KOOS JR outcomes in the TKA subgroup [10, 20]. Force Therapeutics remote PT produced similar KOOS JR, flexion, Timed Up and Go (TUG), and gait speed outcomes compared with outpatient supervised PT through 52 weeks in selected patients [21].

Reported advantages were specific to outcomes rather than uniform across each study. Compared with a paper-based home exercise program, Zhao found better ROM [23]. In diabetic TKA patients, inertial measurement unit (IMU)-assisted telerehabilitation improved selected KOOS domains, strength, and chair-stand performance compared with the same telerehabilitation program without sensor feedback [25]. In addition, Christiansen noted greater daily step counts in the intervention group at 14 weeks, although these differences disappeared by 38 weeks and no differences between groups were observed for any of the standard performance measures [27]. Jung et al. [26] reported better selected stiffness, quality-of-life, and gait ROM measures, while clinical ROM and gait speed were mostly similar [26]. ROMTech Portable Connect reported better early ROM and KOOS JR than outpatient PT, although the study used a retrospective chronological cohort design [11].

### 3.4 Adherence

Adherence was frequently addressed but inconsistently measured. Reported outcomes included exercise completion, compliance percentages, adherence scales, treatment fidelity, and rehabilitation session volume.

Several studies reported higher exercise completion, compliance, or adherence scores in the digital or more digitally intensive group. VERITAS reported higher completion of all prescribed exercises and more PT days per week in the virtual rehabilitation group, and Zhao et al. [23] reported 72% training completion and 90% compliance in the telerehabilitation group [19,23]. ReHub reported higher exercise adherence than standard in-person physical therapy, Sadiq et al. [28] reported a higher proportion of participants with greater than 90% compliance in the intervention group, and multimodal telerehabilitation produced higher Exercise Adherence Rating Scale (EARS) scores than voice-call telerehabilitation [18,24,28].

Other studies reported adherence-adjacent outcomes. ROMTech Portable Connect reported no rehabilitation discontinuations and more home therapy sessions than standard outpatient PT, while the smartwatch/mobile app trial tracked compliance digitally but had internally inconsistent extracted high- and low-compliance subgroup counts [11,20].

There were studies that contained features that aid adherence but did not report straightforward adherence data. GenuSport reports adherence and app acceptance was challenging to measure. Zhou et al. [25] reported high adherence with no specific percentage and Jung developed a home protocol but provided no easily extracted data regarding adherence or compliance [25]. Adherence was comparable in digitally intensive or digital rehabilitation for several studies but was difficult to compare across different studies due to varying measurement techniques.

### 3.5 Pain

Pain outcomes were heterogeneous. Several studies reported no meaningful between-group pain difference or no significant pain interaction over time. VERITAS reported no meaningful pain disadvantage for virtual therapy compared with usual care. UINCARE Home+ showed numerical pain improvement over time but no group-by-time interaction on the numeric rating scale (NRS), and Force Therapeutics remote PT showed no significant NRS differences at 6 or 12 weeks [13,19,21]. Zhao et al. [23] reported no significant visual analog scale (VAS) pain or Short Form-36 (SF-36) bodily pain differences [23]. ReHub [18] reported similar VAS changes and similar WOMAC and EQ-5D outcomes, while Christiansen reported no group difference in WOMAC pain [18,27].

Multiple papers provided evidence for a pain benefit, using a range of measures, comparisons, and study designs. GenuSport demonstrated better short-term rest and activity VAS than standard rehabilitation, and patient-centered telerehabilitation occupational therapy provided significant benefits on the Nottingham Health Profile pain scale after a 12-day follow-up compared with limited control training [17,22]. Sadiq et al. [28] reported improved KOOS pain with a web-based lifestyle modification and sensorimotor training intervention, and multimodal telerehabilitation improved rest and activity VAS more than voice-call telerehabilitation [28]. Correia et al. [15] showed improved KOOS-Pain scores with the digital biofeedback rehabilitation method at 3 and 6 months. ROMTech Portable Connect showed lower VAS pain scores than standard outpatient PT in a retrospective, chronological cohort [11,15].

Pain findings were less consistent than functional recovery because studies measured different pain constructs at different time points and against different comparators. Some reported resting or activity VAS/NRS pain, while others used pain subscales or pain-related components within KOOS, WOMAC, SF-36, Nottingham Health Profile, or total outcome scores.

### 3.6 Satisfaction and Engagement

Satisfaction, usability, patient experience, and engagement were reported inconsistently. Satisfaction or usability results were favorable in the studies that measured them directly, although the instruments and constructs varied by platform. VERITAS reported that 83.3% of virtual rehabilitation participants were promoters, with a net promoter score of 73.8, and patient-centered telerehabilitation occupational therapy improved Canadian Occupational Performance Measure (COPM) satisfaction scores compared with the control group [17,19]. UINCARE Home+ respondents reported high satisfaction scores but also usability problems and installation difficulty, while Force Therapeutics reported satisfaction scores similar to outpatient PT through 52 weeks [13,21].

Several studies reported platform-specific patient experience or usability outcomes. In the mymobility study, patients reported improved preparedness and anxiety-related experience, while daily-activity satisfaction did not differ significantly. Zhao et al. [23] reported 94% app/device satisfaction [10,23]. ReHub [18] reported a high System Usability Scale score, with most users rating the system good or excellent [18]. Correia et al. [15] reported high satisfaction within participants who completed the program; 60% also required caregiver help to use the trackers or app, so the same study captured both satisfaction and support needs [15].

Other studies did not report formal satisfaction or usability outcomes. Tripuraneni did not report patient satisfaction as a distinct outcome [20]. GenuSport did not report satisfaction, and the authors stated that app acceptance and compliance were difficult to assess [22]. Studies that reported adherence, fidelity, app use, session volume, or formal PT utilization alone were not counted as satisfaction or engagement evidence [11,28].

### 3.7 Complications and Readmissions

Complications, adverse events, and readmissions were reported inconsistently. VERITAS reported fewer rehospitalizations in the virtual rehabilitation group, but falls were numerically higher and did not meet noninferiority [19]. Zhao et al. [23] reported one superficial wound infection in each group, no readmissions, and similar adverse-event rates [23]. IMU-assisted telerehabilitation reported similar adverse-event rates between groups, with serious adverse events considered unrelated to therapy [25]. ReHub reported three adverse events in each group, including one medium-severity functional overload event with pain and swelling linked to ReHub; two telerehabilitation participants dropped out after adverse events [18]. ROMTech Portable Connect reported low and similar rates of manipulation under anesthesia, infection, and deep vein thrombosis compared with standard outpatient PT [11].

Several studies had limited safety reporting or did not report readmission outcomes. Tripuraneni reported manipulation-under-anesthesia events but no other complications or readmissions in the extracted data, and GenuSport reported no intraoperative or postoperative complications and no app/tracker technical issues [20,22]. Force Therapeutics reported that no patient in either group met the manipulation-under-anesthesia threshold, but other complication and readmission outcomes were not reported in the extracted data. Christiansen et al. [27] reported four mild activity-monitor skin irritation events and also reported hospitalization and visit counts [21,27].

Torpil and Hong [17] excluded patients with postoperative complications, while Sadiq and Christy did not provide specific adverse-event or readmission counts [16,17,28,29]. Jung et al. [26] reported one patella-fracture exclusion without reporting its relationship to the intervention or readmission outcomes [26]. Reported safety findings were therefore variable and often incomplete, limiting comparison of complications and readmissions across studies.

### 3.8 Cost and Resource Use

Cost and resource-use findings were reported in only a subset of studies and were summarized separately from clinical outcome domains. VERITAS reported lower median 12-week rehabilitation costs for virtual rehabilitation than usual care, with reported mean savings of about $2745 per patient [19]. Force Therapeutics remote PT reported no PT-related travel time or out-of-pocket expenses in the remote group, compared with outpatient copays and travel time in the outpatient group [21]. ROMTech Portable Connect was associated with lower average therapy costs in a traditional Medicare cohort, with reported savings of $2,460.09 per patient. However, the study used a retrospective chronological cohort design, so the cost findings may not generalize beyond that Medicare payment context [11].

Several studies reported utilization or resource use without direct cost analysis. The mymobility smartphone/smartwatch platform reduced formal PT use and reported fewer emergency department visits, but no direct cost analysis was performed, and readmissions were not significantly different [10]. Zhao et al. [23] measured rehabilitation costs and found no significant difference between groups. Correia stated that the digital program was less demanding in human resources but did not report direct cost data [15,23].

Cost-related evidence was limited. Most studies in the main synthesis did not report measured costs, and no study provided a full economic evaluation or formal cost-effectiveness analysis. Studies without measured economic data were treated as contextual rather than cost-effectiveness evidence [22,26,28].

## 4. Discussion

### 4.1 Principal Findings

Overall, functional recovery was generally comparable in selected patients. The clearest support came from studies using usual care or conventional physical therapy (PT) comparators, including VERITAS, mymobility, and Force Therapeutics [10,19,21].

The evidence does not support a broad claim that telerehabilitation is systematically superior to conventional in-person physical therapy. Several studies reported advantages in range of motion (ROM), performance measures, selected KOOS domains, quadriceps strength, or pain-related outcomes, while selected studies also reported better adherence or higher session volume [11,23,25,26]. These advantages depended on the comparator and outcome being measured, because a benefit over written instructions, basic training, or voice-call-only care is not equivalent to comparable outcomes versus supervised outpatient PT [17,23,24].

Secondary and contextual outcomes were more variable. Several studies reported favorable adherence or patient-experience findings, but definitions and measures differed, and pain findings were mixed. Safety and readmission data did not show a consistent increase in major reported events, although interpretation was limited by incomplete reporting and small adverse event counts. Economic evidence remained limited [19,21,23].

### 4.2 Intervention Taxonomy Rationale

The intervention taxonomy was created because the included studies did not evaluate one consistent version of telerehabilitation. A video-based occupational therapy program, a smartwatch-supported pathway, and a clinician-controlled home therapy system all fall under telerehabilitation, but they represent different rehabilitation models with different clinical assumptions [11,17,20].

The taxonomy is intended as a descriptive framework rather than a ranking of platforms. It clarifies what was delivered and what it was compared against, while avoiding the assumption that one feature or platform type caused a specific outcome [19,21,24,25]. Movement measurement or feedback is more directly aligned with ROM, gait, strength, and exercise performance [11,25]. Communication-heavy or behavior-change programs are more aligned with adherence, activity, reassurance, or access [21, 24, 27].

### 4.3 Outcome-Specific Interpretation

In several comparisons of therapy, carefully selected individuals experienced equivalent recovery via structured home-based digitally mediated rehabilitation rather than typical PT, at least within the populations/contexts studied [10,19,20,21]. Any gains observed in ROM, strength, gait, activity or function (patient-reported) need to be evaluated within the context of the comparator/study design and cannot be assumed to be globally superior [18,23,25].

Adherence is clinically important, but it was not measured in one consistent way. Several platforms reported higher exercise completion, adherence scores, compliance, or session volume, suggesting that reminders, progress measurement, and clinician visibility may help patients stay engaged [11,18,19,23,24]. However, due to the variety of measures used, the finding is best viewed as optimistic but not standardized.

Pain outcome did not reflect a pattern similar to functional recovery. Although some papers report similar pain results between digital and control groups, even when functional recovery is equal or better, other papers present favorable pain results, mostly short-term, on interventions including grouped treatment or nonrandomized designs [11,15,17–19,21–24,28]. Such results could be attributed to progression in exercise, reassuring effects of therapy, or perceived comfort with home participation. Yet, there is no direct support for the statement that digital rehabilitation reduces pain following TKA.

Patient experience findings require similar caution. Some studies reported high satisfaction, usability, net promoter scores, or positive patient experience, while others did not include a formal satisfaction measure [10,13,15,18,19,23]. Engagement is not equivalent to satisfaction, and app use, session completion, or messages do not always mean satisfaction was directly measured.

Evidence of safety and readmission is limited. A subset of the studies described similar adverse event (AE) rates. No clear increase in significant reported AEs were detected, although the interpretation of this information is hindered by small numbers of events, poor reporting and some patients with post-op AEs being excluded from data [11,18,23,25].

### 4.4 Clinical Implications

Digital rehabilitation seems to be appropriate for medically fit patients who have been discharged home, are able to follow a plan of exercise, and have access to appropriate technology and/or a caregiver. When such conditions are met, it may help to support the patients’ functional rehabilitation and reduce the burden on patients.

The reviewed interventions were structured care pathways, not stand-alone apps. Models with the strongest practical support included exercise guidance, monitoring, feedback, communication, or clinician review, meaning that a practical program would need explicit instructions, progress assessment, a way for patients to ask questions, and a process for managing concerns when recovery is not progressing as expected [11,13,19,21,23]. Patients with early complications, poor access to technology, limited home support, or difficulty using the platform may still require in-person PT or a hybrid model [15,18,21].

Digital rehabilitation can also alleviate care burden when used as a substitute for some of the face-to-face services. Decisions on cost should be determined based on existing care models, payment mechanisms, or technology cost [23].

### 4.5 Limitations

The first limitation is heterogeneity. Digital rehabilitation varied between app-based exercises, wearable-based pathways, real-time biofeedback applications, clinician-monitored applications, behavior-change interventions, and connected home devices.

The second limitation is inconsistent outcome measurement and reporting depth. Functional recovery was commonly reported, but studies used different combinations of functional outcome measures as discussed in the results. Adherence, satisfaction, engagement, patient experience, safety, readmissions, and resource use were less consistently reported, which limits direct comparison.

A third limitation is the generalizability of these studies. For many, the requirements were having a smartphone, owning a personal computer, access to the internet and e-mail, adequate home support, and access to an individual willing to help. Other studies restricted participation to same-day discharge, diabetic, or Veterans Affairs patients, or only those already technology competent. These limits are relevant to individuals with limited digital literacy, limited internet or device access, or a higher comorbidity burden. [15,20,21,23,25,27].

## 5. Future Research Implications

Clearer definitions of what constitutes a digital rehabilitation model would strengthen future research, including whether such a model supersedes or complements established practice and what outcomes it aims to influence. More consistency across key components would also help platform features, dose of exercise, level of clinician engagement, monitoring routines, communication options, adherence support, and escalation pathways. In addition to a clear description of the clinical comparator, outcomes worth standardizing are function, range of motion, adherence, pain, satisfaction, complications and readmissions. Cost and resource-use data are particularly relevant when digital care is intended to substitute or reduce face-to-face contact [19,23,25].

Future studies would also benefit from reporting digital access, digital competence, caregiver availability, language accessibility, social and economic factors, and reasons for refusal, dropout, or crossover. Longer follow-up would help clarify sustained recovery and safety, and formal economic analyses are needed before stronger economic claims can be made because no study in the main synthesis provided a full cost-effectiveness evaluation [11,19,21].

## 6. Conclusion

Smartphone-based and digitally mediated telerehabilitation appears clinically feasible and useful for postoperative TKA rehabilitation when patients meet selected criteria. Across the reviewed literature, digital telerehabilitation most consistently presented outcomes comparable to or noninferior to those of conventional rehabilitation strategies, while other clinical and contextual outcomes were more variable.

Despite the promising findings, the evidence does not support a broad consensus that telerehabilitation alone is systematically superior to conventional physical therapy. Secondary outcomes were less consistent. Digital telerehabilitation may serve as an adjunct or alternative for selected patients after TKA. Future studies should standardize outcome measurement, lengthen follow-up periods, conduct formal economic analyses, consistently report complications and readmissions, and examine health equity related to device access, technology literacy, and medically diverse or underserved populations.

## Figures and Tables

**Table 1:** Summary intervention taxonomy for digital telerehabilitation after total knee arthroplasty.

Taxonomy model	Included studies	Defining digital features	Most relevant outcome domains
**Real-time biofeedback rehabilitation**	n=6	Platforms used motion, camera, IMU, or sensor feedback during exercise performance, often with app/web exercise guidance and clinician monitoring.	Function, ROM, gait, strength, adherence, pain, safety/usability
**Wearable/sensor-supported rehabilitation**	n=4	Platforms used wearable or activity-tracking devices to support home exercise, activity monitoring, or self-directed rehabilitation, without real-time biofeedback as the dominant model.	Function, ROM, activity, adherence/compliance, patient experience, resource use
**Hybrid digitally supported rehabilitation**	n=5	Programs combined digital exercise materials, telehealth contact, behavior-change support, coaching, video/voice/text communication, or multimodal therapist involvement.	Adherence, pain, activity, patient experience, functional performance, participation
**Clinician-monitored/alert-enabled rehabilitation**	n=3	Systems emphasized dashboard review, remote clinician oversight, uploaded recovery data, smart alerts, automated escalation, or clinician-controlled rehabilitation progression.	Function, ROM, adherence/session volume, satisfaction, safety/readmissions, cost/resource use

**Note:** Categories describe the dominant digital rehabilitation support model and are not intended as a ranking of intervention quality or effectiveness. Outcome domains indicate the domains most directly aligned with the platform features, not proven mechanisms of effect.

**Table 2:** Detailed intervention taxonomy and digital components in included total knee arthroplasty telerehabilitation studies.

Study	Platform/intervention	Core digital components	Comparator	Feature-aligned domains
**Real-time biofeedback rehabilitation (6 studies)**				
Correia et al. [15]	Digital biofeedback home rehabilitation	app/web content; wearable/activity tracking; motion tracking; real-time biofeedback; clinician monitoring; adherence support	Conventional face-to-face home rehabilitation	TUG; KOOS; ROM; adherence/retention; satisfaction; safety; resource use
Bettger et al. [19]	VERA virtual exercise assistant	app/web content; motion tracking; real-time biofeedback; clinician monitoring; messaging/video; adherence support	Usual-care PT	Function; adherence; safety/readmissions; satisfaction; rehabilitation cost
Shim et al. [13]	UINCARE Home+ AR home rehabilitation	app/web content; motion tracking; real-time biofeedback; clinician monitoring; adherence support	Brochure home exercise + weekly phone reporting	Gait speed; ROM; balance; strength; satisfaction/usability; safety
Zhou et al. [25]	Joymotion IMU-assisted telerehabilitation	app/web content; wearable/activity tracking; motion tracking; real-time biofeedback; clinician monitoring; messaging/video	Same app/tablet telerehabilitation without IMU feedback	KOOS domains; strength; chair-stand performance; pain; ROM; safety
Nuevo et al. [18]	ReHub interactive telerehabilitation	app/web content; wearable/activity tracking; motion tracking; real-time biofeedback; clinician monitoring; messaging/video; adherence support	Leaflet/daily exercise plan + domiciliary PT	Adherence; quadriceps strength; active flexion ROM; pain; usability/satisfaction; safety
Jung et al. [26]	eCEN Care wearable motion tracker + smartphone app	app/web content; wearable/activity tracking; motion tracking; real-time biofeedback; clinician monitoring	Conventional self-directed home rehabilitation	Stiffness; gait ROM; clinical ROM/gait; pain; QoL; safety
**Wearable/sensor-supported rehabilitation (4 studies)**				
Tripuraneni et al. [20]	mymobility + Apple Watch self-directed rehabilitation	app/web content; wearable/activity tracking; adherence support	Formal PT	Function; ROM; adherence; formal PT utilization
Baecker et al. [22]	GenuSport sensor game rehabilitation	app/web content; wearable/activity tracking; motion tracking; real-time biofeedback	Conventional standardized rehabilitation/regular PT	Gait speed; early pain; ROM; longer-term function
Alexander et al. [10]	mymobility smartphone/smartwatch care platform	app/web content; wearable/activity tracking; clinician monitoring; messaging/video; adherence support	Standard-of-care rehabilitation	Function; PT utilization; ED visits; preparedness/anxiety; patient experience
Hong et al. [16]	BPMpathway/BPMpro sensor-supported home rehabilitation	app/web content; wearable/activity tracking; motion tracking; clinician monitoring; messaging/video; adherence support	Routine follow-up, education/manual, phone support	HSS knee score; exercise compliance; quality of life; pain-related recovery; follow-up engagement
**Hybrid digitally supported rehabilitation (5 studies)**				
Torpil and Kaya [17]	Client-centered video telerehabilitation occupational therapy	clinician monitoring; messaging/video	Basic home-modification and transfer training	Occupational performance; satisfaction; pain-related quality of life; participation
Christiansen et al. [27]	Telehealth behavior-change program + Fitbit	app/web content; wearable/activity tracking; clinician monitoring; messaging/video; adherence support	Telehealth education attention control + outpatient rehabilitation	Daily steps/activity; rehabilitation fidelity; function; pain; health care utilization
Sadiq et al. [28]	Web-based lifestyle modification + sensorimotor training	app/web content; clinician monitoring; messaging/video; adherence support	Supervised sensorimotor training + written HEP	Balance; proprioception; KOOS pain/function/QoL; adherence; lifestyle self-management
Christy et al. [29]	Virtual rehabilitation and home telerehabilitation protocols	app/web content; real-time biofeedback; clinician monitoring; messaging/video	Conventional PT and alternative digital rehabilitation arms	KOOS; ROM; VAS pain
Aslan et al. [24]	Multimodal WhatsApp telerehabilitation	app/web content; clinician monitoring; messaging/video; adherence support	Voice-call telerehabilitation	Pain; ROM; functional performance; kinesiophobia; quadriceps strength; adherence
**Clinician-monitored/alert-enabled rehabilitation (3 studies)**				
Bradbury et al. [21]	Force Therapeutics remote physical therapy	app/web content; clinician monitoring; messaging/video; smart alerts; adherence support	Electronic perioperative management + outpatient supervised PT	Function; pain; satisfaction; travel burden; out-of-pocket cost; need for in-person PT
Zhao et al. [23]	Smartphone/sensor remote rehabilitation with surgeon dashboard	app/web content; wearable/activity tracking; motion tracking; clinician monitoring; messaging/video; adherence support	Written home rehabilitation + outpatient follow-up	ROM; physical function; adherence; satisfaction; safety/readmissions; cost
Summers et al. [11]	ROMTech Portable Connect home clinician-controlled system	app/web content; connected home device; clinician monitoring; messaging/video; smart alerts; adherence support	Standard outpatient PT	ROM; KOOS JR; pain; rehabilitation cost; session volume/adherence; adverse events

AR, augmented reality; ED, emergency department; HEP, home exercise program; HSS, Hospital for Special Surgery; IMU, inertial measurement unit; KOOS, Knee injury and Osteoarthritis Outcome Score; PROM, patient-reported outcome measure; PT, physical therapy; QoL, quality of life; ROM, range of motion; TKA, total knee arthroplasty; TUG, Timed Up and Go; VAS, visual analog scale. Categories are descriptive synthesis tools and should not be interpreted as proof of superiority or mechanism.

## Data Availability

Data extracted for this review are available as supplementary material.
